# The effect of a clinical decision-making mHealth support system on maternal and neonatal mortality and morbidity in Ghana: study protocol for a cluster randomized controlled trial

**DOI:** 10.1186/s13063-017-1897-4

**Published:** 2017-04-04

**Authors:** Hannah Brown Amoakoh, Kerstin Klipstein-Grobusch, Mary Amoakoh-Coleman, Irene Akua Agyepong, Gbenga A. Kayode, Charity Sarpong, Diederick E. Grobbee, Evelyn K. Ansah

**Affiliations:** 1 School of Public Health, University of Ghana, Legon, PO Box LG13, Accra Ghana; 2 Julius Global Health, Julius Center for Health Sciences and Primary Care, University Medical Centre, PO Box 85500, 3508 GA Utrecht, The Netherlands; 3 Division of Epidemiology and Biostatistics, School of Public Health, Faculty of Health Sciences, University of the Witwatersrand, 27 St. Andrew’s Road, Parktown 2193, Johannesburg, South Africa; 4 Research and Development Division, Ghana Health Service, PO Box MB 190, Accra, Ghana; 5 Regional Health Directorate, Ghana Health Services, PO Box 175, Koforidua, Eastern Region Ghana

**Keywords:** Maternal, Neonatal, Clinical decision-making, Mobile health (mHealth), Text messaging, Ghana

## Abstract

**Background:**

Mobile health (mHealth) presents one of the potential solutions to maximize health worker impact and efficiency in an effort to reach the Sustainable Development Goals 3.1 and 3.2, particularly in sub-Saharan African countries. Poor-quality clinical decision-making is known to be associated with poor pregnancy and birth outcomes. This study aims to assess the effect of a clinical decision-making support system (CDMSS) directed at frontline health care providers on neonatal and maternal health outcomes.

**Methods/design:**

A cluster randomized controlled trial will be conducted in 16 eligible districts (clusters) in the Eastern Region of Ghana to assess the effect of an mHealth CDMSS for maternal and neonatal health care services on maternal and neonatal outcomes. The CDMSS intervention consists of an Unstructured Supplementary Service Data (USSD)-based text messaging of standard emergency obstetric and neonatal protocols to providers on their request. The primary outcome of the intervention is the incidence of institutional neonatal mortality. Outcomes will be assessed through an analysis of data on maternal and neonatal morbidity and mortality extracted from the District Health Information Management System-2 (DHIMS-2) and health facility-based records. The quality of maternal and neonatal health care will be assessed in two purposively selected clusters from each study arm.

**Discussion:**

In this trial the effect of a mobile CDMSS on institutional maternal and neonatal health outcomes will be evaluated to generate evidence-based recommendations for the use of mobile CDMSS in Ghana and other West African countries.

**Trial registration:**

ClinicalTrials.gov, identifier: NCT02468310. Registered on 7 September 2015; Pan African Clinical Trials Registry, identifier: PACTR20151200109073. Registered on 9 December 2015 retrospectively from trial start date.

**Electronic supplementary material:**

The online version of this article (doi:10.1186/s13063-017-1897-4) contains supplementary material, which is available to authorized users.

## Background

The era of the Millennium Development Goals (MDGs) has shown that with appropriate strategies and political will, millions of lives can be improved and saved worldwide [1, 2]. Maternal deaths were halved and under-5 mortality rate (U5MR) declined by more than half over the 25 years of the MDGs [1]. Despite this success, maternal, neonatal and child health care remains a prominent public health concern, particularly in sub-Saharan Africa and Southern Asia where most countries did not attain MDGs 4 and 5 [2–5]. About 13.6 million women are estimated to have died globally from maternal causes between 1990–2015 [2]. An estimated 303,000 of these deaths occurred in 2015; 99% of them occurred in low-and- middle income countries (LMIC) and 66% in sub-Saharan Africa (SSA). Neonatal deaths also accounted for 40% of the eight million childhood deaths in 2010 [6–8]. Ninety-eight percent of these neonatal deaths occurred in LMIC. Though there is evidence of accelerating decline in maternal and U5MR in all regions of the world [2, 3, 9, 10] the rate of decline is uneven among countries [2, 11]. Inequalities still exist in birth outcomes for mothers and their babies globally; the lifetime risk of a woman dying from maternal causes in SSA is 1 in 36 as compared to a lifetime risk of 1 in 4900 in high-income countries (HIC) [2]; neonates born in SSA are six times more likely to die compared to neonates born in HIC [12]. With regards to the decline of U5MR, particularly in the early neonatal period only minor declines have been achieved [9, 11]. It is projected that the global composition of U5MR will continue to shift towards a younger age structure, and that if decreases in child mortality do not focus on neonatal deaths, neonatal deaths will account for about 44.9% of under-5 mortality by 2030 [3].

Maternal and neonatal deaths are caused by a complex interaction of economic, financial, social, cultural and clinical factors [13]. Clinical factors are related to access and quality of antenatal care, skilled attendance at delivery, emergency obstetric care services and postnatal care for neonates. Gaps identified in the quality of care (QOC) given to pregnant women and their newborns include poor quality of clinical decision-making by health providers. Besides knowledge acquired during education in professional training institutions, health providers are known to rely on past experiences, tacit knowledge and intuition, referred to as “mind-lines” [14, 15], in making clinical decisions for their patients. Different categories of frontline providers deliver maternal and neonatal services within and across various health facilities and as such their experiences, intuitiveness and ability to learn from colleagues may vary as shown in differences in risk-taking preferences and attitude towards risk which can lead to significant variations in the way that decisions regarding patient care are made [16]. Reliance on these mind-lines may not be based on empirical evidence, and may affect the QOC rendered to clients. These mind-lines, therefore, cannot be depended upon for sustained QOC which is needed to reduce maternal and neonatal morbidity and mortality.

The use of information and communication technology (ICT) in the form of mobile phones, commonly referred to as mHealth, provides potentially important tools to maximize health worker impact and efficiency [17, 18] and improve service utilization [19] as global efforts to improve maternal, neonatal and child health care intensifies through the Sustainable Development Goals (SDGs) 3.1 and 3.2 already underway [20]. Generally, mHealth interventions are well received by health workers and the community [21]; however, data is limited as to their effectiveness on patient outcomes, efficiency of health systems or their use by health workers [17, 19, 21–24]. Major areas of application of mHealth interventions has been in the area of tools and communication to support health workers, adherence to treatment regime and data collection [21, 23]. Positive evidence for the applicability of mHealth solutions suggest that mobile phones can contribute in reducing the various phases of delay in obtaining help for pregnant women, reducing program cost and improving correct management of patients when used as a decision-making tool [17, 21, 22, 25]. As mobile phone penetration is high even in remote areas in SSA [17] their use by health workers to deliver health care is feasible irrespective of prior education or training in their use [21, 24].

Ghana, a SSA country, is one of the 26 countries whose U5MR contributed to 80% of the world’s childhood mortality in 2013 [3]. The neonatal mortality rate is 29 deaths per 1000 live births with higher mortality rates being reported in rural areas of the country [26–28]. Ghana’s maternal mortality is presently estimated at 319 per 100,000 live births [29]. Clinical causes of persistently high maternal and neonatal mortality identified in Ghana include nonadherence of health workers to clinical guidelines [30, 31]. Though the prevalence of antenatal clinic (ANC) attendance among pregnant women is high in Ghana, skilled attendance at birth is not optimal (about 74%) [27, 32]. Notable barriers to accessing skilled attendance at birth in Ghana include cost, distance, availability of health facilities and attitudes of nurses towards pregnant women [32, 33]. The importance of the QOC given to pregnant women and their newborns for those who opt for skilled attendance at delivery, can therefore not be understated.

To help reduce maternal and neonatal mortality in Ghana, we designed an mHealth intervention – a clinical decision-making support system (CDMSS) facilitating easy access to maternal and neonatal guidelines for routine and emergency obstetric, antenatal and neonatal care for frontline providers of maternal and neonatal care in Ghana. Our main study objective is to assess the effect of CDMSS on the incidence of health facility-based neonatal and maternal mortality.

## Methods/design

### Study design

A cluster randomized controlled trial (CRCT) to evaluate the effect of a mobile clinical decision-making support system on maternal and neonatal mortality and morbidity will be conducted in 16 districts in the Eastern Region (ER) of Ghana. This study will comprise three components: (1) a baseline study to assess the characteristics of the health facilities and outcome measures of interest in study sites, (2) implementation of a CDMSS for 18 months and (3) a sub-study to assess the QOC of maternal and neonatal care services at baseline and at the end of the study.

### Study site

**Fig. 1:**
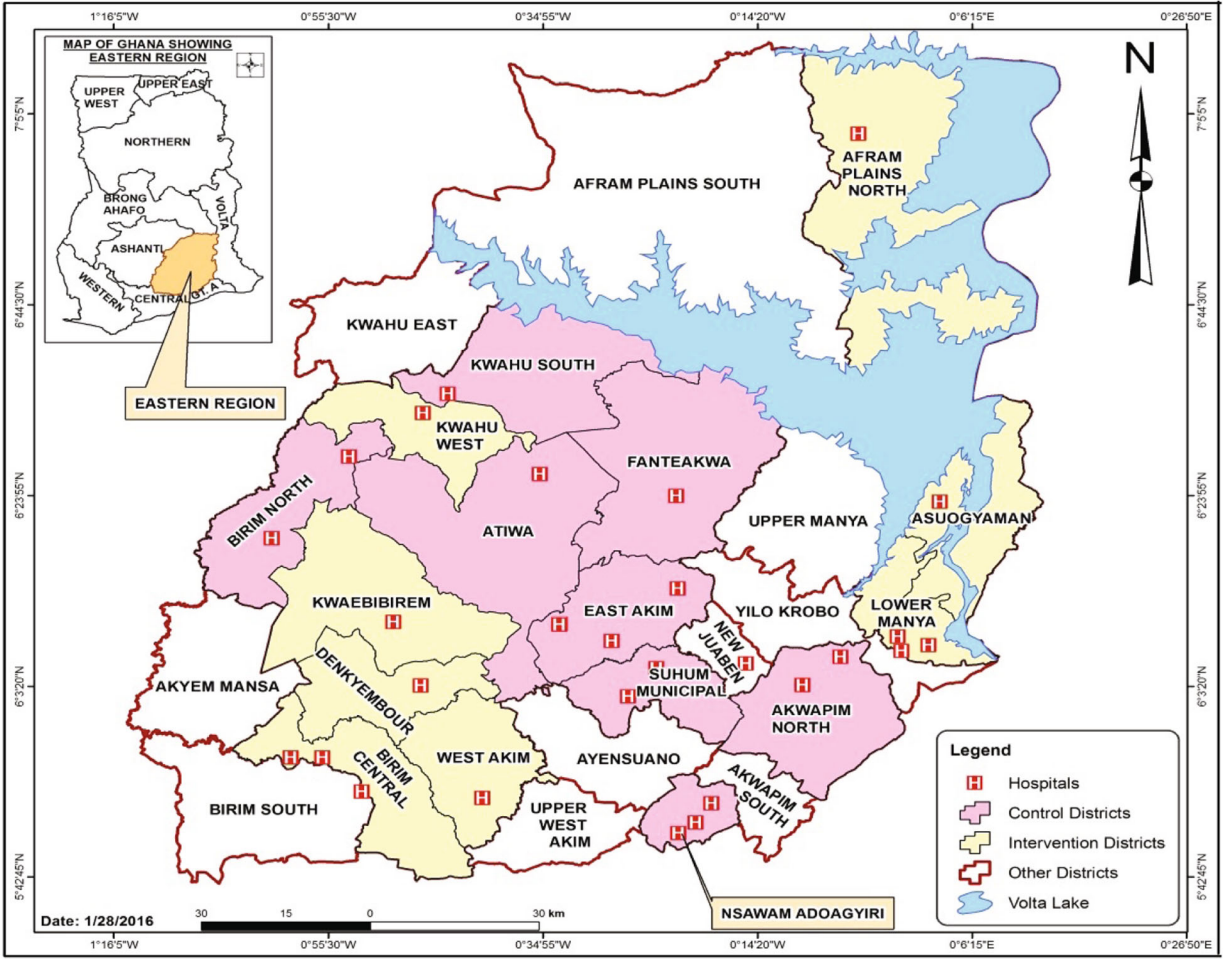
Map of districts in the Eastern Region of Ghana. The districts were defined as cluster units. Sixteen districts fulfilled our inclusion/exclusion criteria. The regional capital, New Juaben Municipal, was excluded from the sampling to avoid selection bias as its regional hospital serves as the highest referral point in the region

The study will be conducted in the ER of Ghana. The ER is the sixth largest region in terms of land area in Ghana (Fig. 1). With an estimated mid-year population of approximately 2.5 million, which is10.7% of the total national population [34], the ER is the third most populous region in Ghana. The region consists of 21 administrative districts with Koforidua in the New Juaben district as its regional capital. The ER is predominately rural in nature with pockets of urban areas in mainly the district capitals. About 40% of its inhabitants reside in 4 out of its 21 administrative districts. The most populous districts are Afram Plains Kwahu North district, West Akim district, Kwaebibirem and New Juaben district in descending order. Agricultural (mainly fish and crop farming) and mining activities are the main stay of economic activities of the region. The estimated growth rate of the ER is 2.0% [35].

Each district comprises a number of subdistricts that form the administrative health subdistricts for the region. There are a total of 250 health facilities, including 31 hospitals in the region, that serve the health needs of the region’s populace [35]. Like other parts of the country, the main categories of health care facilities in the ER are – Community-based Health Planning and Services (CHPS), health centres (HC) and maternity homes and hospitals. At the primary health care level, the CHPS, HC and maternity homes provide services, including maternal and neonatal health services, to the various communities and refer cases to the hospitals. The regional neonatal mortality rate (NMR) was 29 per 1000 live births in 2008 [28]. From 2004 to 2014 the NMR of the ER was estimated as 30 per 1000 live births, showing little change over the period [27]. Presently, the ER ranks sixth in terms of high NMR in Ghana [27]. The pregnancy-related mortality ratio was also 594 per 100,000 live births in 2007 [36]. The ER was selected for this study for two reasons: its high neonatal and maternal mortality rates and because the intervention could not be implemented in the Greater Accra region where it had been designed and piloted [37].

### Cluster selection criteria

The inclusion criteria for cluster selection for this study include the following: (1) The district is located in the ER, (2) The district has expected deliveries of at least 1100/year for the year 2014, (3) The District Health Management Team and the District Hospital Management Team agree to participate in the study and (4) Health facilities within the district conducted at least one delivery in the year 2014. The exclusion criteria for our study are: (1) The district is located outside the ER, (2) The district has expected deliveries of fewer than 1100/year for the year 2014, (3) The District Health Management Team and the Hospital Management Team do not agree to participate in the study and (4) Health facilities within the districts have not conducted at least one delivery during the year 2014.

The year 2014 was selected as the baseline year as the most current data pertaining to deliveries (births) at the time of commencement of the study was for that year. A delivery (births) was a criterion for recruitment as most obstetric and neonatal complications occur around childbirth. Intervention during this period is crucial for survival and health [38].

### Sample size estimation

This study is a superiority trial and has been designed and powered for neonatal mortality to contribute evidence for improved neonatal health care considering the predicted global upward trend in neonatal deaths [3] compared to maternal deaths. Two formulae were applied; the first formula was applied to estimate the required sample size in a randomized controlled trial (RCT) with binary outcome while the second formula was applied to inflate the estimated sample size for a CRCT. Neonatal mortality is the primary outcome, and is currently at approximately 30/1000 live births in the ER, Ghana. Evidence from previous studies including systematic reviews focusing on neonatal care interventions have shown a 23% to 51% reduction in neonatal mortality in settings including LIMC [39–44]. Our intervention will also address neonatal and maternal health care, hence we estimate an effect size of 30% on neonatal mortality with the use of this intervention. Intracluster correlation coefficient (ICC) for neonatal mortality in Ghana has been estimated at 0.0007256 [45]. To detect a 30% decline in neonatal mortality at a power of 80%, a significance level of 0.05 (two-tailed test), with a fixed number of eight clusters in each arm of the study, approximately 1065 patients in each of the 16 clusters will be needed. The first formula is:

$$ m=\frac{n\left(1-\rho \right)}{\left( k- n\rho \right)}, $$

where *m* = number of patients per cluster, *k* = number of clusters in each arms of the study, *ρ* = ICC and *n* = is the number of patients needed to detect this effect in a RCT. The second formula is:

$$ n\kern0.5em =\kern0.5em \theta \left[\frac{\pi_{{}_{\mathbf{1}}}\left(1-{\pi}_{\mathbf{1}}\right)\kern0.5em +\kern0.5em {\pi}_{\circ}\left(1-{\pi}_{\circ}\right)}{{\left({\pi}_{\mathbf{1}}-{\pi}_{\circ}\right)}^2}\right] $$

where *π*_1_ = is the expected proportion of the neonatal mortality at the intervention group after RCT, *π*∘ = is the expected proportion of the neonatal mortality at the reference group after RCT and ϴ is the variance of the two proportions at a power of 80% and significance level of 0.05.

### Randomization

**Fig. 2:**
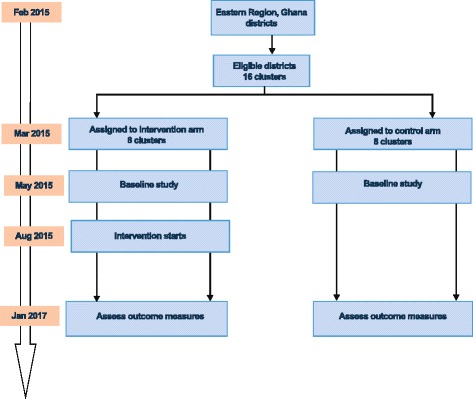
Trial flow chart showing cluster selection, assignment and timelines of the cluster randomized controlled trial (CRCT). Clusters that fulfilled the inclusion and exclusion criteria were randomized into eight control and eight intervention clusters. The CRCT started in August 2015 and ends in January 2017.  Activity, *purple oblong*; Timeline.  *orange oblong*.

The study comprises two study arms – one intervention and one control arm. A cluster unit was defined as a district in this study. Twenty-one districts were, therefore, eligible to be part of the study. Overall, 17 clusters fulfilled the inclusion and exclusion criteria; however, the regional capital was excluded from the selection process to avoid selection bias as its regional hospital is the highest referral point in the region. Sixteen clusters were, therefore, randomized as shown in the trial flow chart (Fig. 2). Cluster randomization was preferred over individual randomization to avoid contamination both at the health professional and client levels, which may occur as a result of social interaction. Randomization was performed by an independent data analyst in order to achieve comparability and avoid selection bias. Randomization was carried out using STATA version 11.0 statistical software. Due to the nature of this intervention, masking was not feasible.

### Sampling of clusters for quality of care study

One well-resourced and one poorly resourced cluster will be purposively selected from each study arm. The selection criteria will be based on the number and mix of health facilities in the district and the midwife to number of deliveries (per annum) ratio in a district. While purposive selection of these study districts does not allow generalizability of findings, application of these qualitative methods provides insight into how and why the intervention worked or not.

### The intervention

The intervention is a clinical decision-making support system consisting of an Unstructured Supplementary Service Data (USSD)-based text messaging of standard emergency obstetric and neonatal protocols to providers on their request, based on the results of a formative study previously conducted in the Greater Accra region [37]. As a reference guideline the national Safe Motherhood Protocol (SMP) [36], an elaborate tool that provides detailed state-of-the-art guidelines for maternal and newborn care, ranging from prenatal care, through antenatal, delivery, postpartum and newborn care, was chosen. A committee of medical experts designed concise and precise protocols with respect to word limits using the USSD system and short protocols using USSD templates have been generated. Access to the USSD platform will be limited to a closed user group (intervention group) who will be provided with subscriber identity module (SIM) cards and cell phones by the research team to avoid contamination. To support the use of the USSD-based text messaging system by health care providers, health care providers in the intervention districts will receive monthly reminders via short messaging service (SMS) on the applicability of the text messaging system for clinical decision-making.

Text messaging based on the USSD system was chosen as a low-cost, easily accessible and instant way of requesting needed information during routine and emergency situations by the health care provider to enhance clinical decision-making. To access the USSD platform, health care workers send a text to a specified short code and this assists in the rapid sharing of the needed information. Access to the USSD platform is free and unlimited. The USSD platform is linked to the general electronic data platform of a telecommunication company whose policy and practice assures 99.99% availability of the general electronic data platform. However, availability of phone reception to assess the network may differ according to location of health facilities.

## Monitoring

Uptake of the intervention will be monitored by the frequency of intervention usage using data about requests made to the USSD collected by the telecommunication company providing technical support for the USSD. All health facilities in the intervention arm will be visited periodically to assess the functionality of the intervention on site. Research assistants (in this case, district health information officers have been recruited) will be trained to supervise data collection activities in the non-hospital facilities in all clusters and to provide updates concerning challenges providers may face with the use of the USSD platform to the research team. The supervisory role of the district health information officers includes ensuring that documentation from the non-hospital facilities is complete and collation of completed data collection forms for submission to the project team. The district health information officers will not make any changes to what is recorded on the completed forms. Thus, they will not assess morbidity or mortality causation.

## Data collection

Data about district and facility characteristics in both arms of the study using a structured questionnaire will be collected. These include human and logistical resources, ANC attendance, number of deliveries, obstetric and neonatal admissions, and primary and secondary outcomes at the baseline and at the end of the study.

The impact of the intervention will be evaluated by extraction of data of outcome measures of interest from the district health information management system-2 (DHIMS-2) database which has been shown to provide reliable estimates of measures [46, 47]. This study will assess the effect of a CDMSS on the commonest causes of neonatal and maternal morbidity and mortality in Ghana. Protocols for diagnosis of these conditions are standardized by the Ghana Health Service across the different categories of health facilities, hence diagnosis across facility type are similar. We assume that health care workers make accurate diagnosis of these common sources of morbidity most of which diagnosis can be made using physical examination and rapid diagnostic tests. The diagnoses made by these health care workers across the different categories of health facilities forms the basis of data entered in the DHIMS-2. Details of all the outcome measures the CRCT seeks to extract from the DHIMS-2; however, covers only hospital data. The non-hospital health facilities report only aggregate data through their district health directorate into the DHIMS-2. Details of outcome measures of interest to the CRCT will, therefore, be collected at the facility level for non-hospital facilities participating in the trial. The diagnosis will be based on what is recorded in the DHIMS-2 or what is recorded in the facility health record books by the health care providers as these diagnoses are expected to follow the standard case definitions for Ghana. Availability of network connectivity in health facilities will be measured during the postintervention evaluation using a Likert scale administered to the health care providers.

The QOC of neonatal and maternal health care services will be assessed by measuring health provider adherence to the standard emergency protocols at baseline and at the end of the study. Provider adherence to these protocols for common neonatal and maternal conditions will be assessed using a checklist based on the SMP in use in Ghana. Qualitative assessment (focus group discussions and key informant interviews among health care workers) as to how the intervention was used and why it produced the effects observed will be conducted in the two intervention districts where the QOC study will be conducted.

**Fig. 3:**
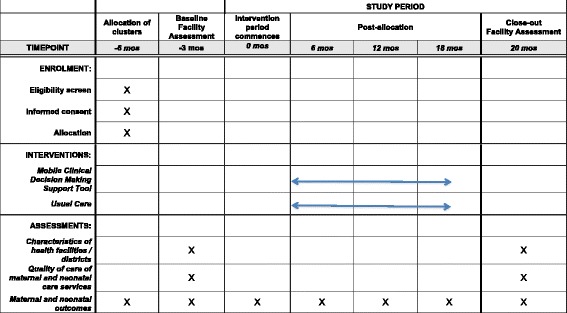
Schedule of enrollment, intervention allocation and assessment using Standard Protocol Items: Recommendations for Interventional Trials (SPIRIT) figure for study protocols

All data will be collected in an anonymous format. Double entry of data collected will be done and data will be handled in accordance with Good Clinical Practice. Figure 3 summarizes the schedule of enrollment and time points for assessments for this study.

## Outcome measures

Institutional neonatal mortality will be considered as the primary outcome measure for this study. For this study neonatal mortality is defined as death of a newborn occurring from birth up to the 28th day of life [12]. Multiple secondary outcome measures pertaining to maternal and neonatal outcomes will also be measured. Aggregate data on primary and secondary outcomes will be extracted from (DHIMS-2) and health facility health record books for non-hospital facilities (Table 1).

**Table 1:** Data sources for outcome measures

Outcome measure	Source of data			
	Hospital	Health centre	CHPS	Maternity home
Number of cases of birth asphyxia	DHIMS-2	Facility	Facility	Facility
Number of cases of low birthweight	DHIMS-2	Facility	Facility	Facility
Number of cases of neonatal jaundice	DHIMS-2	Facility	Facility	Facility
Number of cases of neonatal sepsis^a^	DHIMS-2	Facility	Facility	Facility
Number of cases of PIH/pre-eclampsia/eclampsia	DHIMS-2	Facility	Facility	Facility
Number of cases of postpartum hemorrhage	DHIMS-2	Facility	Facility	Facility
Number of cases of prolonged labor	DHIMS-2	Facility	Facility	Facility
Number of cases of puerperal sepsis	DHIMS-2	Facility	Facility	Facility
Number of cases of neonatal deaths	DHIMS-2	Facility	Facility	Facility
Number of cases of maternal deaths	DHIMS-2	DHIMS-2	DHIMS-2	DHIMS-2
Total number of deliveries	DHIMS-2	DHIMS-2	DHIMS-2	DHIMS-2
Total number of ANC attendants	DHIMS-2	DHIMS-2	DHIMS-2	DHIMS-2

^a^Cord sepsis used as a proxy for neonatal sepsis in this study; *ANC* antenatal clinic, *CHPS* Community-based Health Planning and Services, *DHIMS-2* District Health Information Management System-2, *PIH* pregnancy-induced hypertension

## Statistical analysis

Data analysis and reporting will be in line with the Consolidated Standards of Reporting Trials (CONSORT) Statement guidelines [48]. In this study, randomization was done at the district level and not at the health facility level. All health facilities within a district, therefore, form a cluster. Clusters may differ slightly in their characteristics as can be seen in the larger number of non-hospital health facilities in the control arm (Table 2). We will conduct descriptive analysis of both intervention and reference groups at baseline to explore potential differences in study baseline characteristics of clusters and participants in both study arms. To assess the risk of imbalance in baseline characteristics among the clusters, we will use the c-statistic of the propensity score model of the study. The c-statistic of the propensity score model of the study is considered an appropriate tool to detect baseline imbalance in CRCTs where sample size is large and when a large number of covariates are measured [49]. Any observed imbalance in the baseline characteristics of clusters will be adjusted for in the statistical analysis.

The effect of the CDMSS on primary and secondary outcomes will be analyzed based on the principle of intention-to-treat to minimize over-estimation of the effect of the intervention. Logistic regression will be applied to investigate the effect of CDMSS on all outcome measures considering a potential clustering effect of the CRCT design and adjusting for potential confounders. Results will be reported as relative risks with corresponding 95% confidence intervals. A two-tailed statistical significant level of 0.05 will be used. Among clusters in the intervention arm, descriptive analysis of the availability of network connectivity in health facilities will be done and any significant difference in network availability will be adjusted for using logistic regression. STATA software package [50] and MLwiN software version 2.1 [51] will be employed to handle the analysis.

Descriptive analysis of the adherence of health care providers to standard maternal and neonatal protocols will be done by cluster. Logistic regression will be applied to investigate the association between health provider adherence to protocols and the incidence of maternal and neonatal mortality. Responses from focus group discussions and key informant interviews will be manually transcribed, analyzed and grouped into various themes emphasizing the key convergent and divergent views that explain how and why the health care providers adhered, or did not adhere, to the intervention protocols.

## Expected outcome

**Fig. 4:**
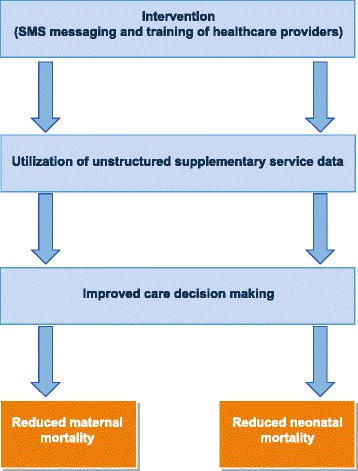
Conceptual framework for evaluating the effect of a clinical decision-making support system (CDMSS) on maternal and neonatal mortality and morbidity in Ghana (2016). The intervention includes training of frontline health workers to utilize the unstructured supplementary service data. The impact of the intervention will be assessed by measuring maternal and neonatal deaths

This intervention is expected to improve clinical decision-making which will lead to a decline in maternal and neonatal deaths (Fig. 4). Neonatal and maternal mortality rates based on the number of live births, neonatal deaths, including perinatal deaths, and the number of maternal deaths in each cluster as well as in each arm will be estimated. These rates estimated will be compared to the rates before the intervention.

## Recruitment and consent of participants

The intervention has been designed for use by frontline providers of maternal and neonatal health care services to support users in making clinical decisions regarding their clients. The impact of the intervention will be measured by extracting data about maternal and neonatal participants who seek care from frontline workers working in health facilities participating in this study (Table 2). Maternal and neonatal participants will be indirectly recruited into this study. Consent to participate in the study was, therefore, sought from the heads of the District Health Management Team, the Hospital Management Team and heads of non-hospital health facilities in the randomized clusters. Prior consultation was held with the Eastern Regional Health directorate team before commencing the research. Written informed consent was sought from all heads of participating district health directorates and health facilities prior to the enrollment of the health facilities into the study. Processes to assure privacy, confidentiality and free choice to withdraw from this study during the conduct of the trial were explained in the Consent Form. Signatures were collected as evidence of consent.

**Table 2:** Number of health facilities participating in the cluster randomized controlled trial (CRCT)

District^a^	Arm	Hospital^b^	Health centres	CHPS	Maternity home
District 1	Intervention	1	1	7	0
District 2	Control	2	6	10	1
District 3	Intervention	1	7	3	0
District 4	Control	1	5	4	1
District 5	Intervention	3	2	9	1
District 6	Control	1	6	7	0
District 7	Intervention	1	3	3	0
District 8	Control	3	4	6	2
District 9	Control	1	3	5	1
District 10	Intervention	3	2	0	1
District 11	Intervention	1	3	2	0
District 12	Intervention	1	8	1	1
District 13	Control	1	7	5	1
District 14	Control	3	6	1	0
District 15	Control	2	4	3	0
District 16	Intervention	1	3	3	1

^a^Districts have been renamed 1 to 16 for anonymity; ^b^Hospitals include private hospitals in clusters; *CHPS* Community-based Health Planning and Services

## Benefits and risk

This intervention is not invasive. Participants are at minimal risk for being part of the study. However, the provider’s standard of health care delivery is being assessed and thus they may feel a little uncomfortable. The researchers will bear this in mind and ensure that this is minimized by assuring providers of anonymity and confidentiality. This study will enable health care professionals to enhance service delivery through access to CDMSS in the case of randomization into the intervention group. Potential benefit to the provider is enhanced service delivery, while to the client the benefit is indirect and long-term. This is because the intervention aims at quality improvement of maternal and neonatal health service delivery.

## Discussion

We have described the protocol for a CRCT to evaluate the effect of an mHealth clinical decision-making support system compared to routine care on maternal and neonatal mortality and morbidity in a context of high maternal and neonatal mortality using the Standard Protocol Items: Recommendations for Interventional Trials (SPIRIT) Checklist (see Additional file 1) [52]. A CRCT is preferred in this kind of intervention to minimize contamination within clusters and optimize scalability of the intervention in a real-life context. This CRCT is being implemented with the support of the regional and local health managers who supervise the work of the frontline health workers. The implementation process of this trial will be well documented. If successful, this CRCT will provide evidence for the use of an mHealth-based CDMSS to reduce maternal and neonatal mortality facilitating the attainment of the SDGs. Lessons learnt from this CRCT can inform recommendations to design and upscale mHealth interventions within the Ghana Health Service system as a whole and in other LMIC, particularly in West Africa. This CRCT will also provide the opportunity to get to know how frontline health workers will use an mHealth intervention to support their clinical decision-making. Data gathered from the requests made to the USSD platform will provide insight into the information needs of frontline health workers of maternal and neonatal contributing to recommendations for training programs for frontline health workers.

Though this study will provide much needed evidence to bridge the knowledge gap about the effect of mHealth intervention on maternal and neonatal health outcomes, the study has some limitations. Firstly, we assume that once the USSD platform is assessed, the information retrieved will be used for action; this may not always be the case. We expect that the assessment of the QOC of maternal and neonatal health care services in the four purposively selected clusters will address this limitation to a large extent. Secondly, there is generally a limitation with regard to network availability, electricity and well-functioning phones in Ghana, particularly in the rural areas; this may affect the ability of health care providers in some remote areas to use the intervention. Thirdly, this study will not assess neonatal or maternal mortality at the community level. The attributable risk of death for neonates born at home compared to those born in health facilities in SSA is estimated to be 21% [53]. The DHIMS-2 database from which data for evaluation of the CRCT will be extracted is a web-based platform built on the District Health Information System-2 (DHIMS-2) open source software. DHIMS-2 serves as a data recording, collection, collation and analysis tool that hosts the entire national health data of Ghana. Data entry into the DHIMS-2 is done using primary data collection tools and standard registers designed for various health services and programs. The DHIMS-2 does not capture community-level health outcomes. The contribution of out-of-facility deaths to maternal and neonatal mortality is, therefore, not captured in the DHIMS-2. The CDMSS has also been implemented as an institutional-based support system to be used by health facilities that contribute data to the DHIMS-2. Thus, community-level data (contribution of the out-of-health facility deliveries to neonatal and maternal deaths) will not be assessed in this study. However, given that 74% of deliveries in Ghana occur in a health facility with a skilled attendant [27], we expect our sample to include the majority of maternal and neonatal clients because clients who self-select to deliver at health facilities are usually followed up in the communities by the health workers. These clients are likely to seek health care services from these facilities. Lastly, there may be concurrent maternal and neonatal health care interventions running at the time of the trial. These interventions could influence the results of our study although they are likely to be independent from the randomization. However, the study team will document all concurrent maternal and neonatal health services occurring in the study sites and carefully interpret our results based on this real-life context.

## Trial status

The trial was registered at ClinicalTrials.gov on 7 September 2015 (trial identifier: NCT02468310) and the Pan African Clinical Trials Registry on 9 December 2015 (trial identification number: PACTR20151200109073). Registration at the Pan African Clinical Trials Registry was done retrospectively after the trial commenced. The recruitment for the trial commenced on 10 August 2015 and is expected to be completed by the end of January, 2017. During the initial 6 months of the CRCT, there were over 2500 requests made to the USSD platform. These requests were made from 94% of health facilities participating in the CRCT.

## Additional file

Additional file 1: SPIRIT 2013 Checklist: recommended items to address in a clinical trial protocol and related documents. (PDF 964 kb)

## Abbreviations

CDMSS: Clinical decision-making support system
CHPS: Community-based Health Planning and Services
CRCT: Cluster randomized controlled trial
DHIMS-2: District Health Information Management System-2
ER: Eastern Region
GCP: Good Clinical Practice
HC: Health centres
HIC: High-income countries
ICC: Intracluster correlation coefficient
ICT: Information and communication technology
LMIC: Low- and middle-income countries
MDGs: Millennium Development Goals
NMR: Neonatal mortality rate
PIH: Pregnancy-induced hypertension
RCT: Randomized controlled trial
SIM: Subscriber identity module
SMP: Safe Motherhood Protocol
SMS: Short messaging service
SPIRIT Checklist: Standard Protocol Items: Recommendations for Interventional Trials
SSA: Sub-Saharan Africa
U5MR: Under-5 mortality rate
USSD: Unstructured Supplementary Service Data
